# Body Fat Mass Assessment: A Comparison between an Ultrasound-Based Device and a Discovery A Model of DXA

**DOI:** 10.1155/2013/462394

**Published:** 2013-02-27

**Authors:** Jean-Claude Pineau, Loïc Lalys, Massimo Pellegrini, Nino Carlo Battistini

**Affiliations:** ^1^Department of Human Evolution, French National Center for Scientific Research, UPR 2147-CNRS, 44 Amiral Mouchez Street, 75014 Paris, France; ^2^Department of Public Health Sciences ,University of Modena and Reggio Emilia, Campi Road 287, 41100 Modena, Italy

## Abstract

*Objective*. To examine measurement of body composition by ultrasound compared with a reference technique:dual energy X-ray absorptiometry (DXA). We evaluated the accuracy of a portable ultrasound-based device in estimating total body fat mass with those assessed by DXA in adult. *Methods*. Body fat mass has been estimated using a portable ultrasound-based device in comparison with a contemporary reference DXA apparatus: the Hologic Discovery A. Anthropometric data has been assessed in order to maximize the output of the software associated with the ultrasound-based device. A cross-validation between ultrasound technique (US) and DXA was developed in this study. Total body fat mass estimated by ultrasound was compared with this DXA model in a sample of 83 women and 41 men. *Results*. Ultrasound technique (US) of body fat (BF) was better correlated with DXA in both women (*r* ^2^ = 0.97, *P* < 0.01) and men (*r* ^2^ = 0.92, *P* < 0.01) with standard errors of estimates (SEE) being 2.1 kg and 2.2 kg, respectively. *Conclusion*. The use of a portable device based on a US produced a very accurate BF estimate in relation to DXA reference technique. As DXA absorptiometry techniques are not interchangeable, the use of our ultrasound-based device needs to be recalibrated on a more contemporary DXA.

## 1. Introduction

Body composition is one of the most important long-term indicator of nutritional status and it is directly related with the health status. In the two compartments model of body composition, body weight (Body Weight, BW) is considered as the sum of body fat mass (BF) and fat-free or lean mass (Fat Free Mass, FFM). Consequently, change in body weight does not give us information on body composition and often generates diagnoses of obesity without considering the relationship between fat mass and lean body mass [1]. Furthermore, body weight alone does not consider adipose tissue distribution while it is well known that the finding of an excessive increase of fat mass, especially in the abdominal-visceral district, may be associated with an increase in cardiovascular risk. DXA represents a reference method for measuring body fat mass. This instrument allows a fast and accurate measurement of the body composition with an exposure to a minimum dose of X-ray radiation. The high cost, the lack of portability, and the relative invasiveness of the technique are limits to the diffusion of this method.

It is known that by ultrasound technique it is also possible to measure subcutaneous fat thickness and to estimate total fat percentage [2, 3]. Until now, the ultrasound technique has often been used for local measurements, in particular to quantify subcutaneous abdominal fat in study of android obesity [4–9], while DXA has rapidly become the reference method for measuring body composition.

We have shown that it is possible to estimate total fat mass using a portable device based on an ultrasound technique. In particular, we have previously tested and validated this new instrument versus DXA (Hologic QDR-4500W) as reference technique in a sample of 89 sedentary subjects of both genders [1]. 

In the course of time, new DXA devices have been developed with new software and shorter data acquisition time. Because of statistically significant differences between measurements obtained with different machines [10–13], users had to carry out new cross-validation studies, with a view to longitudinal followup studies [14–16].

All these reasons lead us to question about the performance of the portable ultrasound-based device, in which measurement of total body fat mass had been estimated in 2002 by comparison with data from a Hologic QDR-4500W DXA instrument. 

According to the fact that total body fat obtained by two different DXA has a significant individual difference, we consider that DXA absportiometry techniques are not interchangeable. For all these reasons, we were led to envisage a new calibration of our portable ultrasound device using data collected with a more contemporary DXA instrument.

## 2. Subjects and Methods

Data was collected on a sample consisted of 83 sedentary women and 41 sedentary men aged respectively 40.1 ± 15.1 and 48 ± 15.3 years at Nancy University Hospital. Only patients who gave written consent after receiving a letter of information on the measurement protocol were included. All patients in good health were recruited according to a wide range of body mass index (BMI) and consequently of total fat mass. In each subject, body composition was measured by DXA radiological examination and by our ultrasound-based device on the same day. All subjects had eaten breakfast in the morning and were properly hydrated before measurement. The reference measures of total body fat mass (BF in kg) were obtained by a Hologic Discovery A (version 12.7.2). This technique, which scans the whole body with an X-ray beam at two energy levels (40 and 100 Kev), is a reference method for measurement of fat mass, lean mass, and mineral content. The subject lies in a supine position for 7 minutes and radiation exposure is very low. The subject's weight is calculated with an accuracy of less than 1%. The results of body composition are available immediately after each examination. 

**Figure 1:**
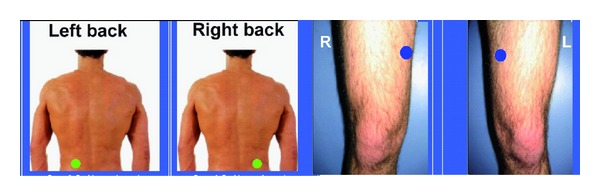
Ultrasound measurement points at the posterior abdominal wall and midthigh (right and left).

Ultrasound measurements were made with a sonographic US BOX in A-mode from Lecoeur Electronique Company (Chuelles, France). Absorption of ultrasound depends on probe depth as well as on the square of the frequency of the waves transmitted. The US can be used to measure the thickness of subcutaneous fat between the skin and the muscle. We selected two preferred anatomical areas: the abdominal areas which are often associated with metabolic risk factors [17–19] and the midthigh area. Subcutaneous fat was located in a horizontal plane with approximately 45° axis vertebral at umbilical level and also at the middle of the knee and the top thigh on the anterior side with a 2.25 MHz linear probe (Figure 1). We used a probe with a 0.75 inches diameter that is the most appropriate in terms of positioning, location, orientation, and contact pressure. Interobserver reproducibility of fat thickness measurements with the ultrasound technique was good, and intraclass correlation obtained by two examiners in the same subjects was greater than 0.98.

Anthropometric measurements, weight, height, and, umbilical waist circumference, were recorded by the same operator using standard anthropometric techniques [20].

### 2.1. Statistics

Fat mass estimation by ultrasound uses a linear regression equation developed from DXA reference value. This regression equation includes the anthropometric characteristics and subcutaneous fat thickness at the midthigh and the back of the umbilical level. Statistical analysis was carried out to compare the fat mass obtained by the ultrasound technique and by DXA absorptiometry using Student's paired *t*-test. The accuracy of the fat mass estimations calculated through the preceding models was evaluated from the determination coefficient *R* ^2^ between true and estimated values of fat mass and the standard error of the estimate (SEE) as described by Lohman [21]. Agreement between body composition estimates was examined by calculating the 95% limits of agreement as described by Bland and Altman [22]. Additionally, potential bias between BF estimates by DXA and the US technique was obtained using residual plots. For all analysis *P* < 0.05 was considered significant. Statistical tests were performed using Statistica software (version 6; StatSoft, Tulsa, Okla., USA).

## 3. Results

The descriptive characteristics of the anthropometric measurements, fat thicknesses measured by ultrasound and the total fat mass (BF in kg) and BF% by DXA of sedentary women and men separately are given in Table 1.

The regression equations used to estimate total fat mass according to Hologic Discovery A are as follows.

For females (*n* = 83)

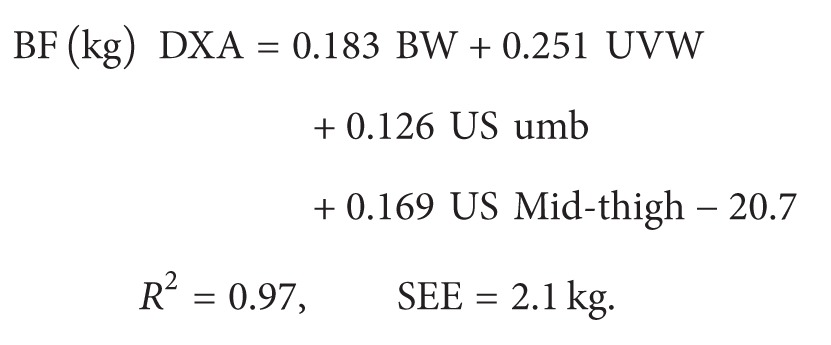
(1)


For males (*n* = 41) 

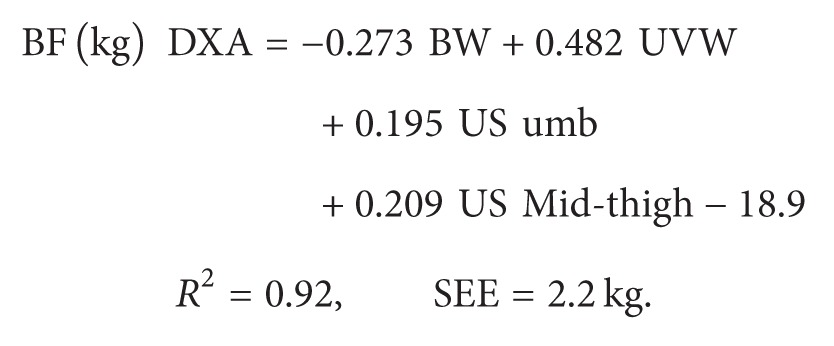
(2)
BW is the body weight (kg); UVW is the umbilical waist-circumference (in mm); US  omb  = (ultrasound thickness of fat at umbilical level (left + right side))/2;  US  mid-thigh = (ultrasound thickness of fat at midthigh (left + right side))/2.

BF% determined by US (31.4 ± 7.0 for women and 20.7 ± 6.4 for men) were no significantly different (*P* > 0.05) than BF% DXA for women (31.5 ± 7.5) and men (21.1 ± 7.2) (Table 1).

Total BF(kg) estimated by US correlated strongly with BF (kg) DXA for females (*R* ^2^ = 0.952, SEE = 2.42) and for the males (*R* ^2^ = 0918, SEE = 2.29). Moreover, BF% obtained by US also correlated with BF% DXA for women (*R* ^2^ = 0.84, SE  = 3.0) and men (*R* ^2^ = .80, SEE = 3.2). 

**Figure 2:**
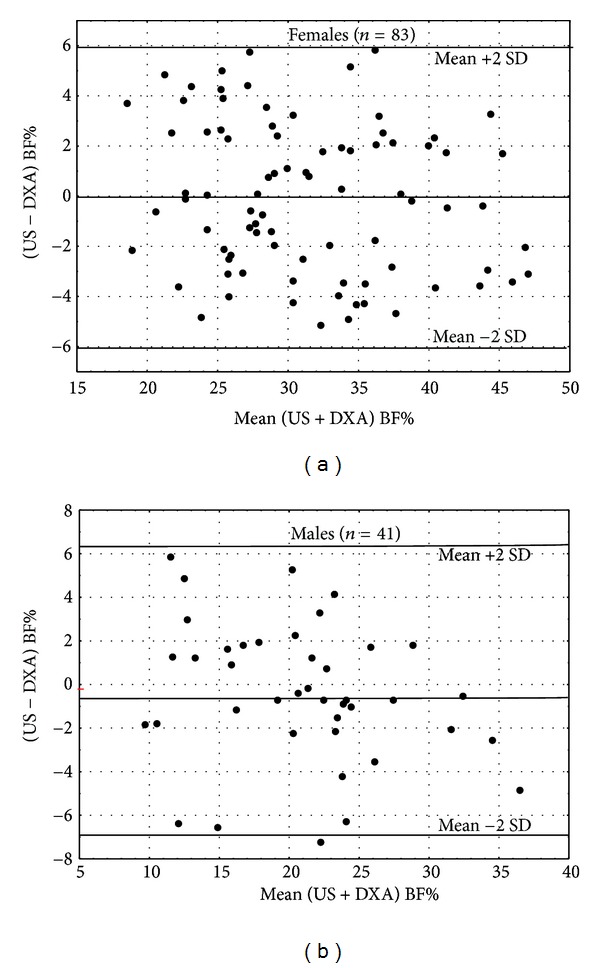
Bland and Altman plots comparing BF% determined by DXA and BF% predicted from US for sedentary females and males.

The Bland et Altman evaluation showed no significant bias between the BF% estimates by US and by DXA with respectively *r* = 0.18, *P* = 0.12 and *r* = 0.25, *P* = 0.20 (Figure 2). The limits of agreement for individual BF% difference between US and DXA were [−5.0% to 5.8%] in females and [−7.3% to 5.8%] in males with a good symmetrically distributed deviations.

## 4. Discussion

The use of a portable device based on ultrasound associated with anthropometry allowed us to measure BF (in kg) and BF% with a high level of accuracy in accordance with the reference method DXA. The US technique that we currently use was first developed in 2002 and based on a DXA reference technique using an Hologic QDR-4500W (version 8.26). Technical advances between 2002 and 2010 have led to changes in the software and in the devices, as the Hologic QDR-4500 W (version 8.26) has been repaved by the Discovery A (version 12.6). These modifications in software and in technique result in a statistically significant difference in individual values between the two apparatus. Most studies comparing body composition measurements between different DXA devices have established that new equation obtained by cross calibration are needed because the apparatus differ in calibration, software, and scan speed [23, 24]. Similar studies have shown that fat mass was significantly higher with the Hologic QDR-4500W than with the Lunar Co Madison, Wi [11]. Standardization of DXA machines is becoming indispensable because of the highly significant differences observed between their results [25]. Generally, machines from the same manufacturers are not interchangeable and cross calibration studies are necessary in order to convent the results of one reference system to the other. Comparison of fat mass in 41 adult women between the Hologic QDR-4500w (version 9.10) and the Discovery A (version 12.6) by cross validation [26] revealed overestimation of means values by the Discovery A compared with the Hologic QDR 4500W. The authors emphasized the need to perform cross calibration for subjects with high fat mass values. 

For all these reason, we have recalibrated the ultrasound device to determine total BF (in kg) and total BF% compared to dual-energy X-ray absorpiometry (DXA), the reference technique. Ultrasound-based device combining US measurements and anthropometric variables proved reliable and accurate for estimating BF and BF% in a sample of dysentery females and males. The results obtained in 83 females and 41 males based on calibration data from physically inactive adults showed a good accuracy compared to the reference DXA measurements. SEE for BF estimates were excellent in females (2.1) and in males (2.2) and good for BF% in females (3.0) and in males (3.2) according to the Lohman's classification [21]. Moreover, the methods described by Bland and Altman [22] were used to examine the level of individual agreement between DXA and US BF%. The 95% limits of agreement found with US ranging from [±6%] for females and [±6.2%] for males denote a good accuracy for the US technique with a good symmetrical dispersion around the mean difference (−0.06 for females and −0.4 for males). Figure 2 also illustrates that there was not a significant US bias for females (*r* = 0.18, *P* = .12) and for males (*r* = 0.25, *P* = 0.20). 

## 5. Conclusion

The ultrasound technique is an accurate method of estimating total body fat mass whatever the reference DXA device used. DXA absorptiometry techniques are not interchangeable as they lead to under- or overestimation of mean fat mass. For this reason, it was necessary to established a cross-validation studies to recalibrate the ultrasound device. The US-based device is portable, noninvasive, nontraumatizing, and harmless. Major disadvantages of DXA include limited availability and high costs, as well as exposure to ionizing radiation, although radiation exposure is low. 

In conclusion, our ultrasound-based device is an indirect technique for measuring BF in adult females and males with good accuracy.

## Figures and Tables

**Table 1:** Characteristics of the study subjects (mean ± SD).

Variables	Females (*n* = 88)	Males (*n* = 41)
Body weight, kg	65.5 ± 18.2	73.5 ± 14.6
BMI, kg/m²	25.0 ± 6.6	24.6 ± 4.5
US thickness, mm		
Umbilical (left + right side)/2	30.1 ± 22.7	35.6 ± 18.7
Midthigh (left + right side)/2	23.8 ± 10.0	18.6 ± 6.9
Circumference, mm		
Umbilical waist	89.9 ± 15.1	91.7 ± 13.9
BF by DXA		
Total BF, kg	21.6 ± 11.1	16.0 ± 7.6
BF %	31.5 ± 7.5	21.1 ± 7.2
BF by US		
Total BF, kg	21.6 ± 10.9	15.7 ± 7.1
BF %	31.4 ± 7.0	20.7 ± 6.4
